# Supplementary material for: A unitary model of auditory frequency change perception
Source: PLoS Comput Biol. 2023 Jan 12;19(1):e1010307. doi: 10.1371/journal.pcbi.1010307 (PMC9876382; doi:10.1371/journal.pcbi.1010307)
Supplement: S1 Text — (PDF) [file pcbi.1010307.s001.pdf]

A unitary model of frequency change perception

Kai Siedenburg[1], Jackson Graves[2], & Daniel Pressnitzer[2]

1Carl von Ossietzky University of Oldenburg, Dept. of Medical Physics and Acoustics, Oldenburg, Germany
2 Laboratoire des systèmes perceptifs, Dépt. d'études cognitives, École normale supérieure, PSL University, CNRS, Paris, France

* kai.siedenburg@uol.de

Supplementary Materials S1

GLME models

Trial-level "down" responses were analyzed using generalized linear mixed-effect (GLME) models as implemented in the `fitglme` class in MATLAB, using a logit link function and a binomial distribution of the response variable. The following provides the model formulas and statistics.

Table A. Exp. 1. Results from the GLME model with the independent variables harmonicity ("harm"), shift type ("type"), and shift size ("size", 1, 2,..., 11), as well as interaction terms (indicated by ":"). Harmonicity and type factors used effects coding (using the following coding scheme: harmonic → 1, inharmonic → -1; SFS → 1, SE → -1). Shift size was coded numerically. The dependent variable ("resp") encoded responses on every trial (down: 1, up: 0). Participants ("part") and the SFS start position of every shift ("start") acted as random effects. The model formula is given by

`resp ∼ 1 + harm * type * size + (1 | part) + (1 | start).`

Variable	β	CI low	CI high	t-value	p-value
intercept	-2.02	-2.32	-1.71	-13.04	$< .001$
harm	-0.26	-0.35	-0.17	-5.73	$< .001$
type	0.01	-0.08	0.1	0.17	0.865
size	0.31	0.3	0.32	45.23	$< .001$
harm:type	-0.52	-0.61	-0.43	-11.39	$< .001$
harm:size	0.06	0.04	0.07	8.49	$< .001$
type:size	0	-0.01	0.01	-0.07	0.944
harm:type:size	0.1	0.08	0.11	14.12	$< .001$

AIC = 62648, $R^2 = .31$.

Table B. Exp. 2. Results from the GLME model with the independent variables harmonicity ("harm"), SFS shift size ("SFS", 1,6,11), first SE shift dummy variable ("SE1", 1 vs. 11), second SE shift dummy variable ("SE6", 6 vs. 11), as well as interaction terms (indicated by ":"). The harmonicity factor used effects coding (harmonic → 1, inharmonic → -1). SFS shift size was coded numerically. Effects coding was used for SE1 (1 → 1, 11 → -1) and SE6 (6 → 1, 11 → -1). The dependent variable ("resp") encoded responses on every trial (down: 1, up: 0). Participants ("part") and the SFS start position of every shift ("start") acted as random effects.The model formula is given by

```
resp ∼ 1 + harm * SFS * SE + (1 | part) + (1 | start)
```

Variable	β	CI low	CI high	t-value	p-value
intercept	-1.28	-1.63	-0.93	-7.17	< .001
harm	-0.39	-0.49	-0.3	-8	< .001
SFS	0.25	0.23	0.26	32.74	< .001
SE1	-0.99	-1.14	-0.85	-13.2	< .001
SE6	0.7	0.57	0.83	10.66	< .001
harm:SFS	0.09	0.08	0.11	12.81	< .001
harm:SE1	0.22	0.07	0.37	2.9	0.004
harm:SE6	-0.06	-0.19	0.06	-0.99	0.322
SFS:SE1	0.06	0.03	0.08	5.23	< .001
SFS:SE6	-0.14	-0.16	-0.12	-14.33	< .001
harm:SFS:SE1	0	-0.02	0.02	0.3	0.765
harm:SFS:SE6	-0.01	-0.03	0.01	-0.95	0.341

AIC = 36416, $R^2 = .45$.

Table C. Exp. 3A. Results from the GLME model with the independent variables shift type ("type", SFS vs. SE), shift size ("size", 1,2,3 for SFS and 1,6,11 for SE), and interaction terms (indicated by ":"). All factors were coded numerically. The dependent variable ("resp") encoded responses on every trial (down: 1, up: 0). Participants ("part") acted as random effects. The model formula is given by

```
resp ∼ 1 + type * size + (1 | part)
```

Variable	β	CI low	CI high	t-value	p-value
intercept	-2.6	-3.0	-2.2	-12.16	< .001
type	-0.92	-1.23	-0.61	-5.78	< .001
size	1.28	1.13	1.42	16.95	< .001
type:size	0.52	0.37	0.66	6.92	< .001

AIC = 7928.9, $R^2 = .33$.

Table D. Exp. 3B. Results from the GLME model with the independent variables SFS shift size ("SFS", 1, 2, 3), first SE shift dummy variable ("SE1", 1 vs. 11), second SE shift dummy variable ("SE6", 6 vs. 11), as well as interaction terms (indicated by ":"). Factor coding analogous to Exp. 2. The dependent variable ("resp") encoded responses on every trial (down: 1, up: 0). Participants ("part") acted as random effects. The model formula is given by

`resp ∼ 1 + SFS * SE + (1 | part) + (1 | start).`

Variable	β	CI low	CI high	t-value	p-value
intercept	-2.25	-2.81	-1.68	-7.78	< .001
SFS	1.01	0.9	1.12	17.76	< .001
SE1	-1.76	-2.13	-1.4	-9.45	< .001
SE6	1.65	1.36	1.95	10.83	< .001
SFS:SE1	0.25	0.09	0.41	3.1	0.002
SFS:SE6	-1	-1.14	-0.86	-13.9	< .001

AIC = 19243, $R^2 = .63$.

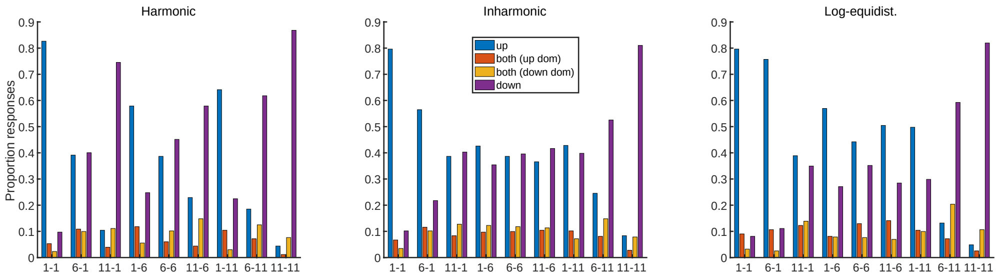

Fig A. Average proportion of responses for sounds with harmonic (left), inharmonic (middle), and log-equidistant partial series (right) for each of the four response categories. Description of the x-axis corresponds to the size of the SFS and SE shift in semitones.

Computational modeling

Table E. R^2 values from Pearson correlation coefficients between raw feature values and average human listeners for AC, CCres, and CCunr features as depicted in Fig. 7. Values in bold font show significant entries according to Bonferroni correction ($\hat{\alpha} = \alpha/9 = 0.05/9 = 0.0056$.)

		SFS	SE	SFS-SE
AC	Harmonic	**.97**	.01	**.88**
	Inharmonic	**.83**	.00	**.75**
	Log-equidist	.93	.99	.30
CCres	Harmonic	**.75**	**.96**	.58
	Inharmonic	**.83**	**.87**	**.93**
	Log-equidist	.61	.99	.60
CCunres	Harmonic	.00	**.91**	.06
	Inharmonic	.39	**.89**	.52
	Log-equidist	1.0	.88	.66

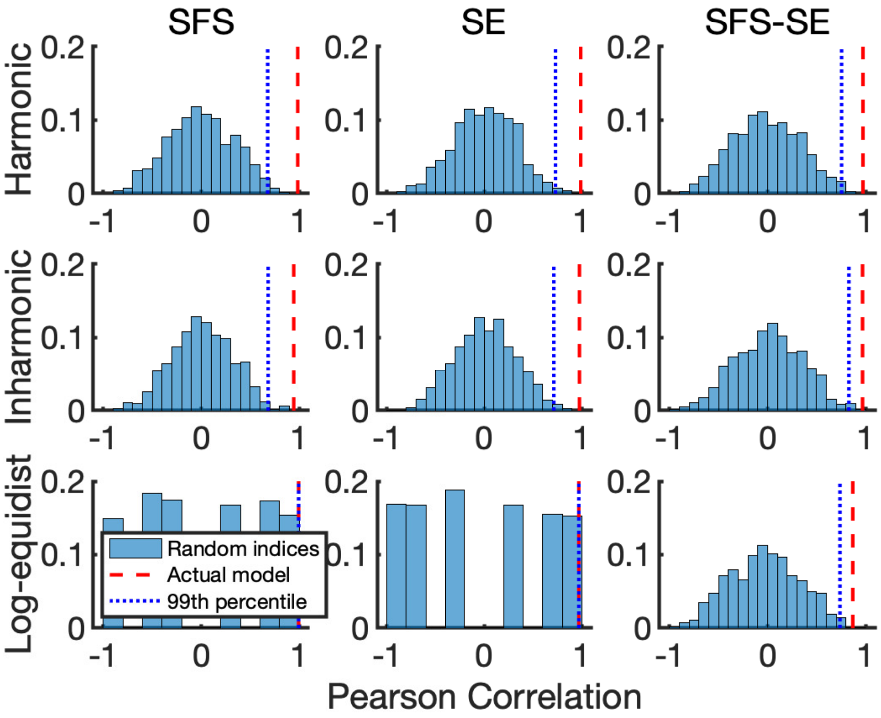

Fig B. Histogram of correlations values for model fitted with randomly permuted shift factor indices, measured over 1000 random permutations. Also shown are the actual correlations with the model for unperturbed indices (dashed red line) as well as the 99th percentile of the empirical distributions (blue dotted line).

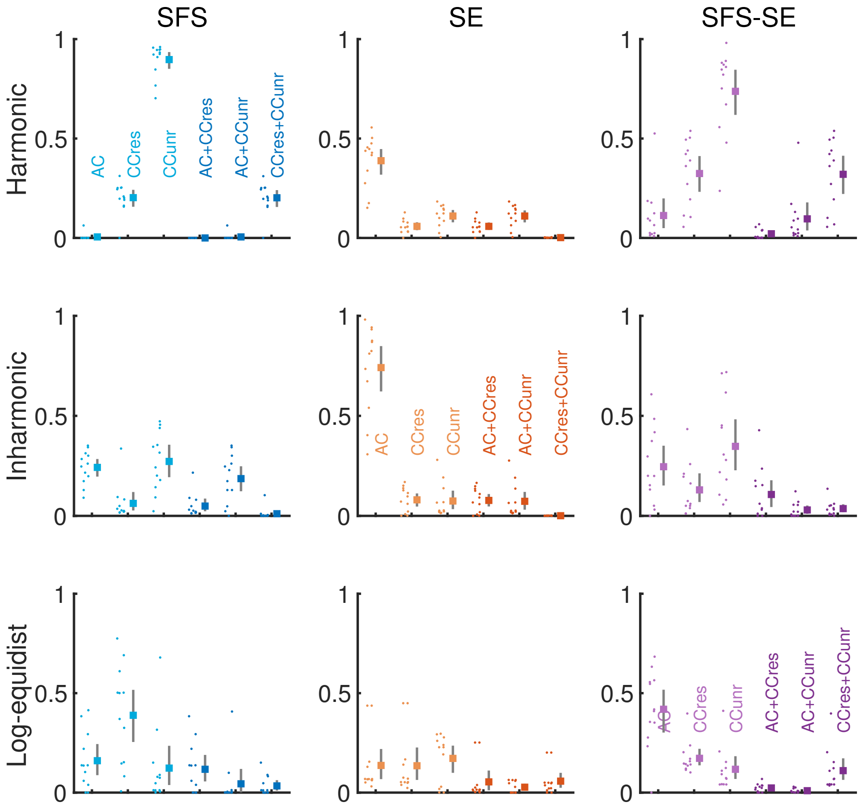

Fig C. Distribution of paired differences of R^2 values (displayed on the y-axis) between three-dimensional complete model (AC+CCres+CCunres) and lower-dimensional incomplete model variants (x-axis, see panels on the diagonal for labels). Panels sorted according to fine structure type (rows) and acoustic shift dimensions (columns). Dots correspond to differences for individual participants, square symbols to mean; error bars indicate boostrapped 95% confidence intervals. Of particular interest are the two-dimensional shifts displayed on the rightmost side. Here, it is visible that for the harmonic case, the fit of the AC+CCres model is indistinguishable from the full model. For the inharmonic case, however, the AC+CCres variant exhibits poorer fit (CIs non-overlapping with zero). That is, the full model is necessary to account for the general case of harmonic and inharmonic sounds.

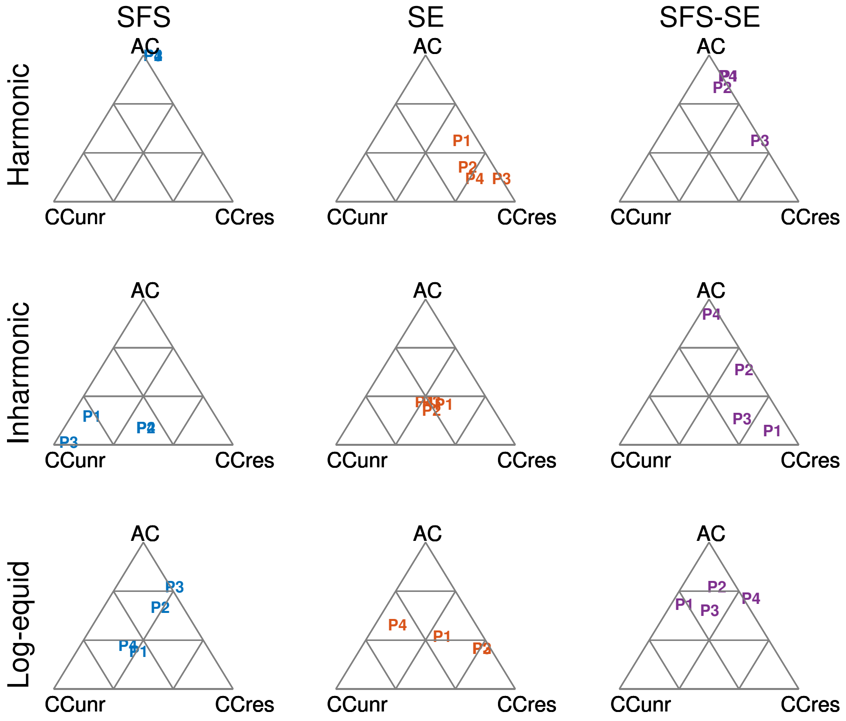

Fig D. Individual weights from the four participants P1-P4 that concluded all three experiments. The location of the letter Px corrresponds to the model weight.

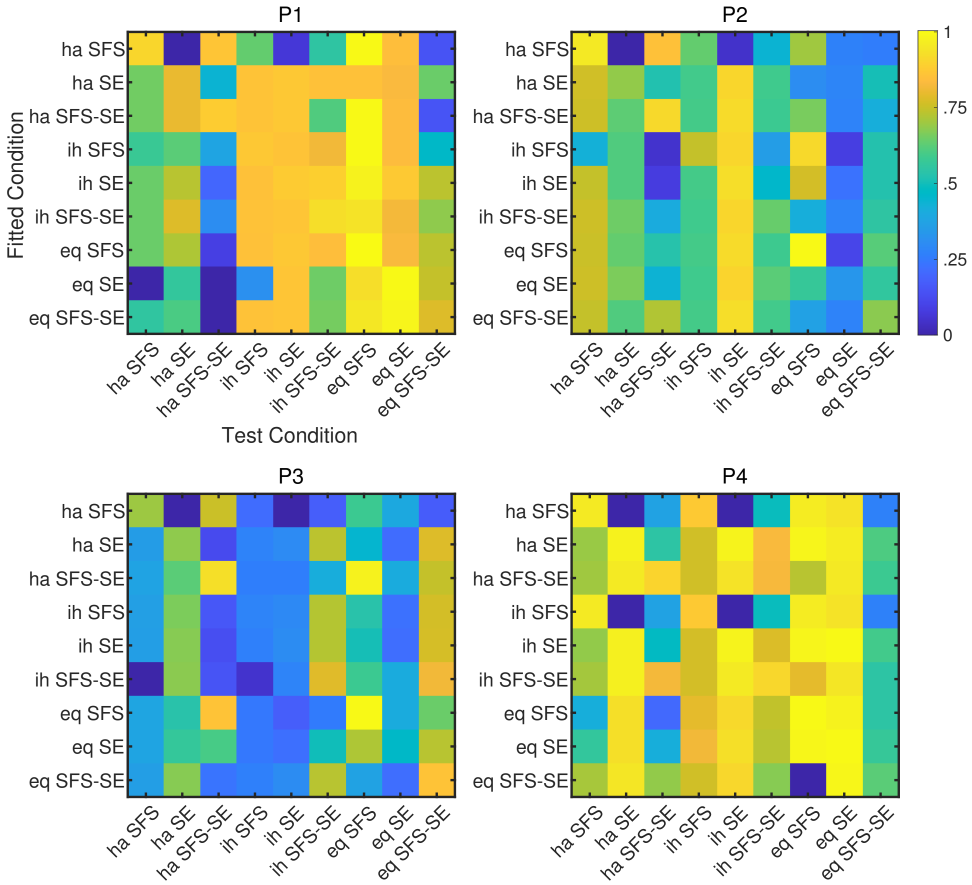

Fig E. Generalization of model predictions across conditions. The figure depicts R^2 values via color coding for individual model predictions from the four participants who completed all three experiments. Models were fitted on conditions depicted on the y-axis and tested on conditions depicted on the y-axis.
